# Supplementary material for: XDH-1 inactivation causes xanthine stone formation in Caenorhabditis elegans which is inhibited by SULP-4-mediated anion exchange in the excretory cell
Source: PLoS Biol. 2025 Sep 24;23(9):e3003410. doi: 10.1371/journal.pbio.3003410 (PMC12479013; doi:10.1371/journal.pbio.3003410)
Supplement: Attachment — Submitted filename: 250718_FormattedResponse copy.pdf [file pbio.3003410.s009.pdf]

UNIVERSITY OF SOUTH DAKOTA
SANFORD SCHOOL OF MEDICINE

SANFORD RESEARCH

July 18, 2025

Dear Luke,

Thank you for coordinating these 3 insightful reviews of our manuscript, *XDH-1 inactivation causes xanthine stone formation in C. elegans which is inhibited by SULP-4-mediated anion exchange in the excretory cell.* We were very excited to see that the reviewers thought our work to be rigorous, novel, and impactful. Reviewer #1 commented on the quality of our data, "I find this work to be thorough and genetically rigorous." Reviewer #2 notes that, "The work is novel" and "…the paper is well-written, and the experimental design is sound." Finally, Reviewer #3 states that our work, "nicely demonstrated several phenotypes highlighting metabolic dependence."

While positive, each reviewer raised several major and minor issues in their evaluations. Please find our point-by-point response to each issue below. In our revisions we have performed new experiments, incorporated new data, and altered text and figures per reviewer suggestions. The incorporation of these changes has dramatically improved the quality, rigor, and clarity of our work and we believe the revised manuscript will be of great interest to the broad readership of *PLoS Biology.*

Thank you very much for your time and consideration.

Sincerely,

Kurt Warnhoff, Ph.D.
Associate Scientist, Pediatrics and Rare Diseases Group, Sanford Research
Assistant Professor, Pediatrics, Sanford School of Medicine, University of South Dakota
Phone: +1 (605) 312-6469 Email: kurt.warnhoff@sanfordhealth.org

Point-by-Point response to reviewers.

Note, the line number callouts refer to the manuscript version that *does not* display track changes.

Reviewer #1:

Issue 1: *I find this work to be thorough and genetically rigorous. However, some of the data interpretation was confusing and seems to ignore simple hypotheses for more complex ideas. Also, the paper lacks an integrated model that accounts for all of the data presented so I found it very hard to interpret the overall significance.*

Response: We have dramatically altered our Results, Discussion, and Figures to highlight the simpler hypotheses alluded to here by Reviewer #1. Furthermore, we have overhauled our model figures (**Fig. 1A and 7**) to better account for all the genes, pathways, and data displayed in our manuscript. Please see our responses to subsequent issues for specific details of how our manuscript has evolved during peer review.

Issue 2: *A model that integrates the data would be very helpful to readers and should be included.*

Response: We have generated a new streamlined model (**Fig. 7**) suggesting a mechanism, based on our data, by which *sulp-4* loss of function might promote xanthine stone formation.

Issue 3: *It seems to me that a simpler model than the 'osmotic stress' model proposed by the authors is that whole animal sulfur levels, derived in part from cdo-1/cth-2 catabolic pathways, increase when sulfur excretion is blocked in sulp-4 mutants. Excess sulfur drives stone formation. Loss of cdo-1/cth-2 lowers sulfur generation and blocks stone formation. Was this model tested and disproven in some way?*

Response: We agree with the model proposed by the reviewer and believe this issue has arisen due to a failure in communication on our part. As an extension of this model, we propose that sulfate accumulation, driven by CTH-2/CDO-1 and inactivation of *sulp-4* causes an osmotic imbalance which promotes xanthine stone formation. We have heavily modified the text (Abstract, Results, and Discussion) and generated a new model Figure (**Fig. 7**) to better communicate this model.

To experimentally test the impact of *sulp-4* on sulfur homeostasis, we have established a new collaboration with the lab of Dr. Martina Ralle. Together, we measured sulfur (in addition to Na, K, Fe, Zn, Mn, Cu, and Mo) in wild-type and *sulp-4(rae319)* mutant animals using inductively coupled plasma mass spectrometry (ICP-MS). We did not observe any difference in sulfur content between wild-type and *sulp-4(rae319)* mutant animals, demonstrating that *sulp-4* mutants do not accumulate sulfur. However, we point out that, while state of the art, ICP-MS does not discriminate between various sulfur species in animals (i.e. sulfurs found in sulfate, sulfite, cysteine, methionine, etc.). Thus, we cannot exclude the model that *sulp-4* mutant *C. elegans* accumulate sulfate and experience a concomitant decrease in one of these sulfur-containing metabolites that maintains whole animal sulfur at consistent levels. It is also possible that sulfate sulfur might be a tiny fraction of the total animal sulfur pool when compared to the highly abundant sulfur containing amino acids like cysteine and methionine. Thus, any change in sulfate concentration may be obscured by the high background of these common metabolites. These data and discussion points have been incorporated into **Fig. S5** and the Results (**lines 283-295**).

As the reviewer points out, we propose that sulfate (produced by CTH-2/CDO-1) is promoting xanthine stones. We speculate that sulfate accumulation may drive osmotic imbalance, promoting conditions in the intestinal lumen that facilitate xanthine stone formation. This model is supported by our observation that loss of *osm-8* (a key modulator of osmotic homeostasis) also promotes xanthine stone formation when *xdh-1* is inactive. New data (**Fig. 6A**) also supports the concept that *sulp-4* mutant animals are experiencing osmotic stress as they accumulate sodium when compared to the wild type. High sodium (hypernatremia) is a driver of dehydration, a key risk factor for kidney stone formation. These new data are discussed in the Results (**lines 318-323)** and Discussion (**lines 424-440**). For additional comments, please also see our response to **Issue #8.**

Issue 4: *What do the authors mean by the sulp-4 mutant causing osmotic stress? Do they mean that blocking sulfate transport in the excretory cell increase overall solute levels enough to osmotically stress the animal, which activates the osmotic stress response, like exposing animals to hypertonic stress or knocking out osm-8? If so, osm-8 type mutants have a very striking osmotic stress resistance (Osr) phenotype. Do sulp-4 mutants have an Osr phenotype like osm-8?*

Response: Yes, our model is that loss of *sulp-4* causes sulfate accumulation (mediated by CTH-2/CDO-1) promoting osmotic imbalance (see response to **Issue #3**).

Additionally, the reviewer suggests testing osmotic stress resistance in *sulp-4* mutant animals. We performed this experiment using wild-type and *osm-8(n1518)* animals as controls. We found that *sulp-4* mutant animals displayed NaCl tolerance like the wild type. As expected, *osm-8(n1518)* mutant animals displayed dramatic NaCl resistance. These new data are displayed in **Fig. 6B** and referenced in the Results (**lines 348-349**).

Issue 5: A further prediction of this osmotic stress model is that growing animals in a hypertonic environment should also drive stone formation. Was that tested? If so, is it cdo-1/cth-2 dependent?

Response: This is an excellent experiment that we had considered previously. We tested whether supplemental NaCl might induce xanthine stones in *xdh-1* mutant *C. elegans.* Unfortunately, we found that *xdh-1* mutant animals were exquisitely sensitive to supplemental NaCl, preventing us from performing this experiment. In our revised manuscript, we have quantified this phenotype and incorporated the data into the new **Fig. 6B.** These data are also highlighted in the results (**lines 324-330**). Interestingly, rats lacking XDH are also hypersensitive to a high salt diet, suggesting this observation is conserved (DOI: 10.1016/j.isci.2022.104887). These results have opened a new line of research that we are pursuing in the lab currently and prompted us to take a genetic approach to testing the role of osmotic stress in the formation of xanthine stones (i.e. using the *osm-8(n1518)* mutation).

Issue 6: *Can xanthine stone formation occur in vitro? If so, that could be an interesting setting in which to directly test the sulfur hypothesis.*

Response: We are not familiar with a method for evaluating xanthine stone formation *in vitro.* However, development of this assay could aid in future mechanistic studies of xanthine stone formation.

Issue 7: *Is there any evidence that C. elegans accumulates taurine as an osmolyte in response to osmotic stress? Also, where taurine is used as an osmolyte in mammalian cells, it is usually accumulated via a membrane transporter, not so much by biosynthesis.*

Response: We appreciate this feedback. We think that our focus on taurine in our original manuscript distracted from our data and proposed model. As we do not have any evidence that *C. elegans* accumulates taurine, we have removed any claims about a role of taurine in xanthine stone formation throughout our manuscript. Although, we still mention that taurine is a product of CDO-1 (**line 359)**.

Issue 8: *Osm8 mutants contain high levels of glycerol, which creates an osmotic driving force for water reabsorption. Could the effect of osm-8 simply be due to dehydration of the intestinal lumen, which would increase hypoxanthine concentration and promote stone formation?*

Response: Yes, this is a possible mechanism by which "osmotic imbalance" may be promoting xanthine stones. For *osm-8* inactivation, this imbalance could be driven by glycerol accumulation. For *sulp-4* inactivation, this imbalance could be driven by sulfate accumulation downstream of CTH-2/CDO-1. We have elaborated on this in the Discussion (**lines 424-440**).

Issue 9: How could hypoxanthine accumulate to high enough levels in the intestinal lumen to form stones, given that the worm defecates and flushes the intestinal lumen every 45 seconds? Are xdh-1,, sulp-4, or other stone-inducing mutants defecation-defective?

Response: To test the hypothesis that the defecation cycle may limit the formation of xanthine stones, we performed a new experiment using the *aex-5(sa23)* mutation. *aex-5(sa23)* causes defects in anterior body-wall muscle contraction (aBoc) and waste expulsion (Exp), critical aspects of the defecation cycle. Interestingly, *aex-5; xdh-1* double mutant animals did not display an increased penetrance of xanthine stone formation. These new data have been added to **Fig. S3A** and the Results section (**lines 246-253)** These results suggest that defects in defecation alone are not sufficient to promote xanthine stone accumulation.

Issue 10: *The pictures of the excretory cell in figure S3 are not of sufficient quality to determine whether or not sulp-4 alters cell morphology. The cysts are notable in exc-5 and are clearly not present in sulp-4. However, I can not determine if the excretory cell tubules are fully extended in either WT or sulp-4.*

Response: We have replaced the images in **Fig. S3** with a new, higher-resolution study of wild-type, *exc-5,* and *sulp-4* mutant *C. elegans* expressing *Pvha-1::GFP* (*qpIs11*). We have also amended our results with more precise language regarding the absence of cysts in *sulp-4* mutant animals. We do not feel our imaging analyses are sufficient to comment on excretory cell tubule extension and have included this caveat in the results section (**lines 235-245)**.

Issue 11: *The qPCR data are somewhat concerning and do not support the conclusion on line 404-405 that sulp-4 causes increased expression of gpdh-1 and hmit-1.1. The authors cannot claim this as evidence while simultaneously claiming the changes are not statistically significant. Unfortunately, I think the 1 hour starvation prior to RNA isolation is to blame here, given that gpdh-1 and hmit-1.1 are well established by multiple labs to be strongly upregulated by osm-8 and other Osr mutants, as well as hypertonic stress, a finding that was not reproduced using the authors starvation protocol. If the authors wish to make this claim, the experiment should be repeated, probably without the 1 hour starvation.*

Response: We have repeated the mRNA quantification of *hmit-1.1* and *gpdh-1* (normalized to *act-1*) on cDNA derived from wild-type, *sulp-4(rae319),* and *osm-8(n1518)* animals. This time, the animals from which the mRNA was isolated were not washed for 1 hour in buffer M9 (as in our original manuscript). All other methods and statistical analyses were identical. This experiment was performed on 3 new biological replicates for each *C. elegans* strain. We again observed highly variable and non-significant increases in *hmit-1.1* and *gpdh-1* mRNA in both *sulp-4* and *osm-8* mutant animals when compared to the wild type (**Reviewer Response Fig. 1A,B,** below). It again *appears* that these mRNAs are accumulating, but in our hands the variability of the accumulation prevents statistical significance. Given that we saw no significant impact on mRNA induction by removing the 1-hour washing step (**Reviewer Response Fig. 1C,D,** below), we have opted to use our original qPCR datasets as we have data from more biological replicates. As the reviewer suggests, we have removed any claim that *sulp-4* inactivation activates the transcriptional hyperosmotic stress response. We cannot explain why we observe such variable and non-significant induction of *hmit-1.1* or *gpdh-1* mRNA in *osm-8* mutant animals. We assume this is caused by some experimental variable for which we have not accounted. For transparency, we have left the data in **Fig. S6A,B** but have made no claims regarding these data given that this is no longer a major focus of our manuscript (**lines 343-357**). Where appropriate, we have cited the peer-reviewed manuscripts demonstrating the induction of these osmotic response genes.

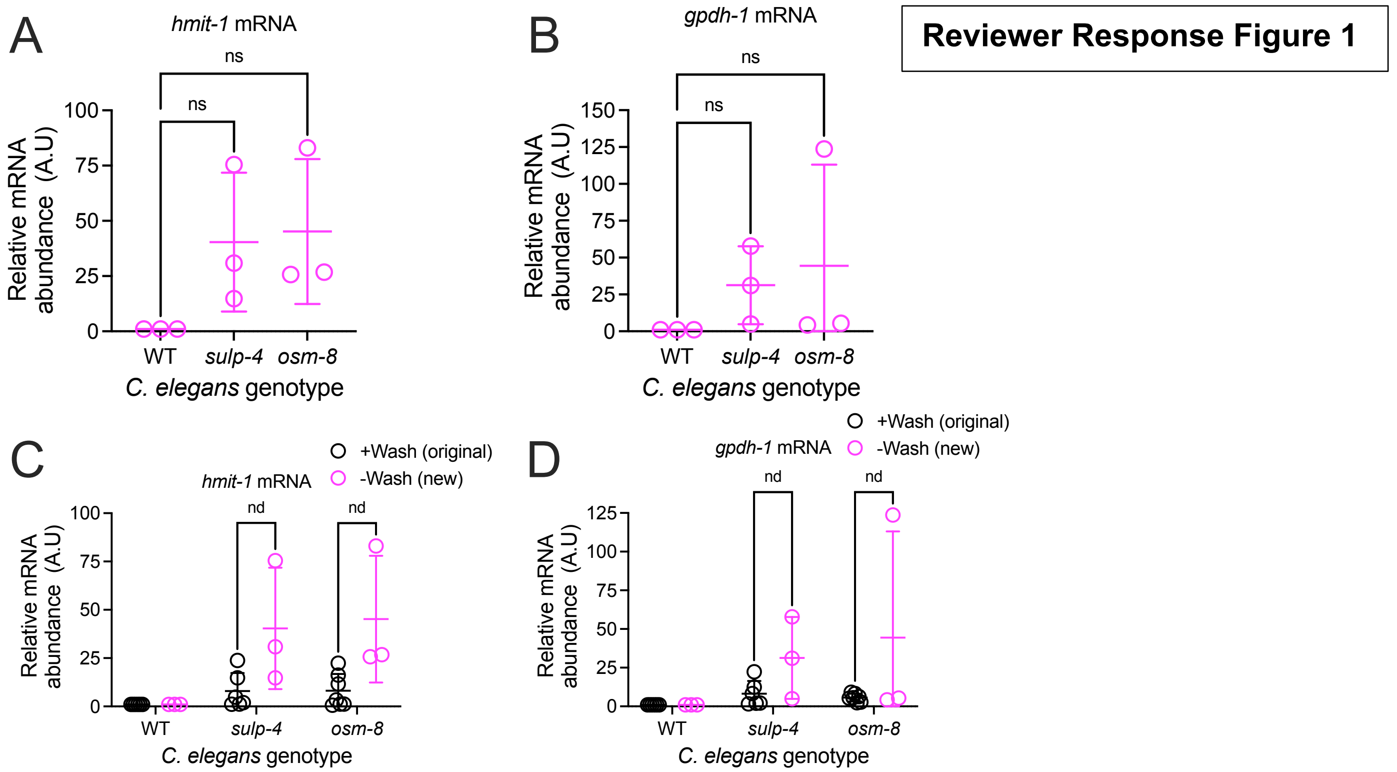

Reviewer #2:

Issue 12: *The work is novel; however, it seems incomplete as there is no description of the impact of stone formation on any aspect of worm biology. While it is interesting that the authors have documented stone formation in C. elegans for the first time, it is unclear how pertinent this system is. For example, the authors state that the role of SLC26 transporters in stone formation has already been documented. Also, are a large percentage of xanthinuria cases in humans due to unknown genetic mutations that could be illuminated by genetic screens in C. elegans? As the formation of stones is linked to dietary intake, this seems unlikely. Perhaps, this interpretation is completely inaccurate and if so, it would then be critical for the authors to stress the impact of this model beyond the discovery of xanthine stones in C. elegans.*

Response: As the reviewer states, we have not assessed the impact of xanthine stone formation on worm biology. Here we are simply using the development of xanthine stones as a visible and viable readout for defects in purine homeostasis (xanthine degradation or excretion). Determining the physiological impact of xanthine stones on worm biology (lifespan, brood size, fertility, etc.) is an interesting future direction that we hope to pursue.

We believe our system is pertinent as it has revealed that failures in sulfate transport (*sulp-4* loss of function) dramatically impacts the likelihood that an *xdh-1* mutant animal will form a xanthine stone. We are not attempting to identify new genetic mutations that cause xanthinuria- these are known as is stated by the reviewer. Rather, we are trying to identify and characterize pathways acting in parallel to mitigate stone formation even when *xdh-1* is completely inactivated. This tells us about systems that may be acting as a failsafe to prevent the catastrophic formation of xanthine stones. We have modified this phrase, *“Surprisingly,*

only a small percentage of Moco-deficient and xdh-1 mutant C. elegans developed xanthine stones, suggesting additional parallel pathways for limiting stone formation. To identify novel regulators of xanthine stone accumulation, we performed an unbiased chemical mutagenesis screen for mutations that enhanced the penetrance of xanthine stone formation in xdh-1 mutant animals" (**line 67-70**) in the background to more clearly spell out the goals of the work.

Major Revisions:

Issue 13: *Figure 1A should be replaced with a schematic of Moco synthesis, moc genes, and interactions with cth-2 and cdo-1. Please describe cth-2 and cdo-1 and their interaction with Moco when these genes are first introduced, not in the discussion. Fig 1A fits better with the data presented in Fig 3 and should be moved there.*

Response: As suggested by multiple reviewers, we have altered our model in **Fig. 1A** to incorporate pathways for Moco synthesis and import, sulfur amino acid catabolism (CTH-2, CDO-1, and SUOX-1), and purine catabolism (PNP-1 and XDH-1). We have also added descriptions of *cth-2* and *cdo-1* when these genes are first introduced (**see lines 91-93**).

Issue 14: *Quantifying worm length indicates that the mutants presented in Fig 2D, Fig 3D, and Fig 5C are shorter than N2 72 hours post-L1 plating, but it does not directly address their larval stage. If the authors want to state that larval development is affected, they must quantify the number of animals that reach the L4 stage 48 hours post-L1 plating.*

Response: To address this concern, we quantified the percentage of animals 72 hours post L1 that i) have not reached the L4 stage, ii) are currently L4s, or iii) are young adults. We have added these new data to the Results in multiple locations where we reference **Fig. 2D, Fig. 3B,** and **Fig. 5C.** These new analyses demonstrate that *sulp-4* inactivation delays larval development.

Issue 15: *Only one biological replicate for Sup Fig 2B is shown. Published results from experiments should be performed in triplicate at a minimum.*

Response: The requested replicates have been performed for **Fig. S2B**.

Issue 16: *Along with including more biological replicates for Sup Fig2B, demonstrating that overexpression of another SLC26 homolog does not rescue the stone formation in xdh-1;sulp-4 will strengthen the specific function of sulp-4 in contrast to general disruption of anion transport due to overexpression of sulp-4.*

Response: We are not perfectly clear on what is being suggested here. We do not have any evidence that overexpression of *sulp-4* causes a general disruption of anion transport as stated by the reviewer. Our only data with *sulp-4* overexpression demonstrate that it rescues the enhanced xanthine stone formation displayed by *xdh-1; sulp-4* double mutant animals **(Fig. 4**). For SLC26 homologs, the only scenario where we see enhanced xanthine stone formation is with *sulp-4* loss of function. This is why we queried deletions in other *sulp* genes (**Fig. S2B**).

We could imagine testing whether other SULP proteins, expressed under the *sulp-4* promoter, might rescue xanthine stone formation in *xdh-1; sulp-4* mutant animals. For example, one could engineer a construct such as *Psulp-4::SULP-1::GFP* and repeat the experiments displayed in **Fig. 4C.** This would inform us about the interchangeability of these related transport proteins and likely reveal whether these transporters share

common substrates (or not). However, this could potentially be complicated by what membrane each SULP is targeted to (apical vs. basolateral). These experiments are interesting and would help systematically define the functional relationship between various SULP proteins and SULP-4. However, we do not feel they are necessary to support the conclusions presented in our current manuscript.

Issue 17: *The number of haploid genomes screened in the xdh-1 enhancer screen and the total number of mutations and complementation groups should be provided.*

Response: Over multiple rounds of EMS mutagenesis, we surveyed a pool of ~100,000 haploid genomes. However, because this screen was clonal, we sought to increase the likelihood of identifying mutations that enhanced xanthine stone formation in an *xdh-1* mutant background. To do this, we first isolated F2 animals that developed a xanthine stone and cloned them onto a fresh NGM petri dish seeded with *E. coli* OP50. We reasoned this would enrich the clones we screened for potential mutants of interest. We ended up cloning ~600 stone positive F2s and from that pool isolated 5 loss-of-function alleles of *sulp-4.* This information has been added to the Methods section (**lines 559-564)**.

Issue 18: *Provide a model figure tying the expression and function of sulf-4, osm-8 and xdh-1 to stone formation in the intestine.*

Response: We feel that future studies are still required to tie the expression pattern and functional site of action for SULP-4, OSM-8, and XDH-1 into a cohesive model explaining stone formation. This is because we do not yet know the functional site of action for OSM-8 or XDH-1 with respect to xanthine stone formation. As suggested by multiple reviewers, we have generated a simplified model figure (**Fig. 7).** We have also expanded our explanation of this model in the Results (**lines 367-375).**

Other issues/questions:
Issue 19: *What are the three other Moco-requiring enzymes and their function?*

Response: These details have been added to the Background section (**Lines 112-113).**

Issue 20: *Has xdh-1 been shown to require Moco for its enzymatic function, or is this just assumed based on sequence homology to XDH? Were is xdh-1 expressed?*

Response: That *C. elegans* XDH-1 requires Moco for enzyme activity is an assumption based on strong sequence homology to human XDH. Our BLAST results using *C. elegans* XDH-1 as the query aligning to the human proteome identify human XDH as the top aligned hit with an E value of 0.0 and 47% amnio acid identity across 99% of the protein.

C. elegans XDH-1 is expressed in neurons, intestine, and the excretory cell (https://doi.org/10.15252/embr.201948671). We have added this information and citation to **lines 118-119.**

Issue 21: *re319 and re320 have the same mutation and display different penetrance of stones. Is the annotation correct in Fig 2A?*

Response: This is correct. In fact, we know these are separate mutagenic events as they emerged in completely distinct rounds of EMS mutagenesis of the parental *xdh-1* mutant strain. We suspect the variability in penetrance is the result of a background mutation(s). Importantly, after outcrossing 4 times, the strain carrying *sulp-4(rae319)* still displays highly penetrant xanthine stones when fed a Moco- diet. These data also

match the highly penetrant stone formation caused by *sulp-4(rae334);* a null mutant produced using CRISPR/Cas9. Thus, we believe USD1013, which harbors *sulp-4(rae320),* may contain a background mutation that limits the formation of xanthine stones. However, we have not experimentally explored this possibility.

Issue 22: *To state that xdh-1 and sulp-4 act in parallel, one needs to determine if there is a significantly higher penetrance in stones in an xdh-1;sulp-4 double compared to the singles. Figure 2B does not do this, as the single mutants are not shown. This statement is confirmed by piecing together data from multiple figures (Fig 1B, Sup Fig 2A, and Fig 2B), but it needs to be presented more clearly.*

Response: We have added xanthine stone penetrance data for *sulp-4(rae319)* single mutant animals to **Fig. 2B.** As suggested, this more clearly demonstrates that *xdh-1* and *sulp-4* are indeed acting in parallel to limit the formation of xanthine stones.

Issue 23: *Where is pnp-1 expressed, and how does this relate to xdh-1, xanthine, and stone formation?*

Response: PNP-1 is expressed in the intestine and head neurons (https://doi.org/10.1371/journal.ppat.1009350). We have added this information and citation to **lines 189-190.** It seems likely to us that purine catabolism driven by PNP-1 and XDH-1 is occurring in the intestine. This is based on the published localization patterns and our discovery that xanthine stones form in the lumen of the intestine. However, there are no functional studies (tissue-specific rescue for example) of these enzymes related to a metabolic phenotype (including xanthine stones). Understanding the spatiotemporal dynamics of purine metabolism (using xanthine stones as a readout) is an exciting avenue that we hope to pursue in the future.

Issue 24: *Can exogenous supplied taurine reverse the suppression of stone formation observed in xdh-1;sulp-4;cdo-1 mutant? This would strengthen the connection to osmotic stress.*

Response: We have not performed this experiment. However, as mentioned in our response to **Issues #3 and 7**, we are de-emphasizing the role of taurine in our model and highlighting sulfate as the likely cause of osmotic stress. We focus on sulfate as it is a known substrate of SULP-4 and product of CTH-2/CDO-1 downstream of methionine and cysteine catabolism.

Our lab does have experience in supplementing *C. elegans* cultures with cysteine which is a valuable experimental metabolite as it is the product of CTH-2 and the substrate for CDO-1. If a metabolite downstream of cysteine (like sulfate) is enhancing the formation of xanthine stones, then we would expect cysteine to promote xanthine stones in *cth-2; xdh-1; sulp-4* triple mutants but not in the *xdh-1; sulp-4; cdo-1* triple mutants. This is exactly what we show in our new **Fig. 5B.** These data demonstrate that a metabolite downstream of cysteine and CDO-1 is promoting xanthine stones in *xdh-1; sulp-4* mutant animals. We argue this metabolite is sulfate, the direct substrate of SULP-4. This new experiment is detailed in the Results, **lines 296-306** and **Fig. 5B.**

Issue 25: *Where is osm-8 expressed? A quick search indicates that it is expressed in the hypodermis. How does this relate to stone formation in the intestine and the function of sulp-4 in the excretory cell?*

Response: *osm-8* is indeed expressed in the hypodermis (https://doi.org/10.1371/journal.pgen.1001267). We have added this information and citation to **lines 333-334**. As mentioned in our response to **Issue #23**, we are very interested in the spatial organization of the players involved in our manuscript (XDH-1, PNP-1, OSM-8,

CTH-2, CDO-1, and SULP-4). We imagine that understanding site(s) of action for these proteins will be extremely informative to help learn how a metazoan maintains metabolic homeostasis and senses imbalances to respond accordingly. Yet, we do not know the site of action for these proteins (other than SULP-4) with respect to xanthine stone formation. We feel to intelligently comment in this area would require many transgenic rescue experiments that are beyond the scope of the current manuscript.

Reviewer #3:

Issue 26: *Method explanations: Stone counts / %: Methods should be more clear as to what a positive stone count is and how "% penetrance" is calculated. It seems that even one crystal counts that worm as positive. The % penetrance then comes from the number withing that group that have "a crystal".*

Response: The reviewer's interpretation of our methods is correct. To clarify this, we have added a more explicit definition of "% penetrance" to our methods in **lines 611-628.**

Issue 27: Method explanations: C. elegans larval delay: it is implied but not stated (especially for the non- C. elegans reader) that larval length is used as a surrogate for larval delay. Please reference or explain more overtly

Response: This issue was also raised by Reviewer #2. Please see response to **Issue #14.**

Issue 28: *Discussion: Why is xanthine accumulating in xdh-1; sulp-4 animals if SULP-4 is a chloride and sulfate transporter? What is the connection? For example, xanthine is typically degraded to uric acid (urate) which can also form stones. Is there evidence of uric acid crystals forming? Or is metabolism further in C. elegans? (e.g., allantoin, urea, NH3 ?). Considering this further metabolism may implicate why defective sulp-4 results in a back up of xanthine. Why would removing sulfate from the metabolic steps prevent an accumulation of xanthine?*

Response: Many of these issues were raised by other reviewers. Please see our response to **Issues #3, 4, and 8.**

We do not have any evidence of uric acid crystals forming. In fact, we would anticipate uric acid to be deficient in our *xdh-1* mutant animals as uric acid is the product of XDH-1. We only observe substantial autofluorescent stones when XDH-1 activity is reduced via mutations in *xdh-1* or reductions in dietary Moco. Additionally, we do not know if purine metabolism goes further in *C. elegans* than uric acid. A quick BLAST search does not identify a urate oxidase homolog encoded in the *C. elegans* genome; however, follow-up studies (perhaps metabolomic) would be required to test this hypothesis further.

Issue 29: *Discussion: Is there an effect of worm size per se on xanthine crystal formation? Smaller worms would have a tendency to for crystals more readily due to smaller spaces for xanthine to accumulate, Figures 2 and 5 would seem consistent with this interpretation. Are there reasons why these would be dissociated?*

Response: We do not see an obvious correlation between worm size and xanthine stone formation. Importantly, the size reductions presented in Fig. 2 and 5 are not directly comparable to the animals used in our assays for xanthine stone formation. Fig. 2 and 5 show animals that were 72 hours from L1. Many of these animals were not adults and in fact were delayed in their development (see response to **issue #14**). For experiments with xanthine stone formation, animals were not synchronized at the L1 stage but were selected as L4s and only assayed during adulthood. In our experience, xanthine stone formation is not driven by size

but by time. We rarely observe a xanthine stone during larval development, and that only occurs in our enhanced *xdh-1; sulp-4* mutant condition. Xanthine stones tend to occur as adult animals age, presumably as they accumulate more xanthine. We make this point in **Fig. S1** (**line 1048-1050**) where we had to age our *xdh-1* mutant animals to identify enough stone positive animals to image. We have modified the Methods to clarify the distinct animal preparation that goes into our "animal length" studies vs. our "xanthine stone penetrance" studies.

Issue 30: *Supplemental figures. These 4 figures would seem appropriate in the main text as each has important data that are presented in Results.*

Response: For clarity of presentation, we prefer to keep these figures in the Supporting Information. We believe that the key plot points of our story are reflected in the main body figures.

Minor.
Issue 31: *Suggest using "xanthine crystal" rather than "xanthine stone".*

Response: We prefer to use the term xanthine stone rather than crystal. We acknowledge that the xanthine masses we are observing in *C. elegans* are microscopic and perhaps more comparable to crystals in human urine. But from the perspective of the worm, these xanthine accumulations are quite large, more akin to a stone (relatively speaking).

Issue 32: *Microscope / filter setting for xanthine crystal autofluorescence? Are these FITC/GFP settings or other?*

Response: Xanthine stones were visualized and imaged using EGFP BP (FITC/Cy2) HC Filter Set. We have added this information to the Methods (**lines 643-644**).

Issue 33: *Check background on stone types. There are more recent assessments than 1954.*

Response: We have updated our references and explicitly added a more modern reference providing case studies of xanthine urolithiasis to **line 380** (DOI: 10.1016/j.urology.2005.10.057)**.**

Issue 34: *Is size of xanthine crystal significant? For example, Supp Fig 1 shows that osm-8;xdh-1 crystals are much smaller than those observed with xdh-1;sulp-4.*

Response: We presume that the size of a xanthine stone is reflective of the amount of xanthine from which the stone is composed. Thus, we would anticipate that *xdh-1; sulp-4* mutant xanthine stones would have more xanthine than their *osm-8; xdh-1* or *xdh-1* counterparts. We have added this interpretation to our Results section (**line 159-161**). We further acknowledge this using genetic language by stating that, "Loss of *sulp-4* enhanced the expressivity of the xanthine stone phenotype displayed by *xdh-1* mutant animals."

Issue 35: *In testing for xanthine crystal penetrance (Supp Fig 2), why are sulp-3 and sulp-6 missing? If viable strains are not available, this should be stated around line 196-197.*

Response: *sulp-3* and *sulp-6* homozygous mutants have been reported to be inviable, thus they were excluded from our analyses. We have clarified this point in **lines 214-215.**

Issue 36: *Perhaps a Table of C elegans genes and functions would help a more general reader.*

Response: In lieu of providing a new table, we have expanded our model **Fig. 1A** to include the genes/proteins used in this study. This was also suggested by other reviewers.

Issue 37: *No institutional association for Ashley Van Asma.*

Response: Thank you for catching this oversight. We have added her affiliation to the revised manuscript (**line 4**).
