# Supplementary material for: XDH-1 inactivation causes xanthine stone formation in Caenorhabditis elegans which is inhibited by SULP-4-mediated anion exchange in the excretory cell
Source: PLoS Biol. 2025 Sep 24;23(9):e3003410. doi: 10.1371/journal.pbio.3003410 (PMC12479013; doi:10.1371/journal.pbio.3003410)
Supplement: Attachment — Submitted filename: 250828_ReviewerResponse.pdf [file pbio.3003410.s010.pdf]

Point-by-Point response to reviewers.

Reviewer #1:

Issue 1: *However, I do have one remaining concern related to the last bit of data on gpdh-1 and hmit-1.1. The authors use gpdh-1 and hmit-1.1 deletion alleles to test the role of glycerol and myo-inositol in stone accumulation. Loss of gpdh-1 alone does not reduce steady state glycerol levels (Lamitina et al 2006). The authors would need to delete both gpdh-1 and gpdh-2 to reduce glycerol levels (and even that only reduces high salt induced glycerol accumulation by ~60%). I am not aware of any data examining myo-inositol levels in C. elegans, much less if loss of hmit-1.1 function reduces myo-inositol levels. This is also complicated by the presence of 3 hmit genes in worms, which could also be involved in regulating myo-inositol levels. So statements about the specific roles of glycerol, myo-inositol, or other osmolytes (or lack thereof) are not warranted. The authors should change their conclusion to address these limitations.*

Response: We greatly appreciate the reviewer pointing out this weakness in the interpretation of our genetic results from **Figure S6C.** We have modified the relevant results section to specify that our genetic conclusions only pertain to loss of *gpdh-1* or *hmit-1.1* and highlight that these experiments are confounded by the existence of additional genes homologous to both *gpdh-1* and *hmit-1.1.*

Reviewer #2:

Issue 2: *My only comments regard the author's statement that for Fig 2D, 3B, and 5C, the previous data using worm length as a proxy for larval development has been replaced with quantification of time to reach the young adult stage.*

1) The data in these figures has not been replaced
2) The figure legends have not been updated to reflect this change
3) The methods section still includes a description of how the worm length has been measured.

This should be corrected before publication.

Response: We have added new figure panels to **Fig. 2, 3, and 5** to include graphs of the larval development data that was previously only mentioned in the text of the results. We have also updated the figure legends and methods where appropriate. Thus, we have graphically represented both our original length measurements as well as the larval development assay that was added during the initial round of revision.
